# Physicochemical Characterization of FRET-Labelled Chitosan Nanocapsules and Model Degradation Studies

**DOI:** 10.3390/nano8100846

**Published:** 2018-10-17

**Authors:** Stefan Hoffmann, Christian Gorzelanny, Bruno Moerschbacher, Francisco M. Goycoolea

**Affiliations:** 1 Institute of Plant Biology and Biotechnology (IBBP), Westfälische Wilhelms-Universität Münster, Schlossplatz 8, 48143 Münster, Germany; stefan.hoffmann@wwu.de (S.H.); moersch@uni-muenster.de (B.M.); 2 Department of Dermatology and Venerology, University Medical Center Hamburg-Eppendorf, 20246 Hamburg, Germany; c.gorzelanny@uke.de; 3 School of Food Science and Nutrition, University of Leeds, Leeds LS2 9JT, UK

**Keywords:** chitosan, nanocapsules, degradation, FRET, protein corona, bioimaging

## Abstract

Sub-micron o/w emulsions coated with chitosan have been used for drug delivery, quorum sensing inhibition, and vaccine development. To study interactions with biological systems, nanocapsules have been fluorescently labelled in previous works, but it is often difficult to distinguish the released label from intact nanocapsules. In this study, we present advanced-labelling strategies based on Förster Resonance Energy Transfer (FRET) measurements for chitosan-coated nanocapsules and investigate their dissolution and degradation. We used FRET measurements of nanocapsules loaded with equimolar concentrations of two fluorescent dyes in their oily core and correlated them with dynamic light scattering (DLS) count rate measurement and absorbance measurements during their disintegration by dissolution. Using count rate measurements, we also investigated the enzymatic degradation of nanocapsules using pancreatin and how protein corona formation influences their degradation. Of note, nanocapsules dissolved in ethanol, while FRET decreased simultaneously with count rate, and absorbance was caused by nanocapsule turbidity, indicating increased distance between dye molecules after their release. Nanocapsules were degradable by pancreatin in a dose-dependent manner, and showed a delayed enzymatic degradation after protein corona formation. We present here novel labelling strategies for nanocapsules that allow us to judge their status and an in vitro method to study nanocapsule degradation and the influence of surface characteristics.

## 1. Introduction

Nanomedicine has been gaining traction over the past few decades, particularly in drug delivery, diagnosis, and medical imaging research. Many applications are currently being investigated, in clinical trials, or have already been commercialised. Pharmaceutical formulations clinically approved include liposomes [1,2], albumin nanoparticles [3], and polymeric micelles [4]. The advocated advantages of these systems are enhanced drug delivery options and feasibility of other therapeutics that are normally challenging to deliver owing to free molecules issues [4], prolonged circulation time [3,5] or specific targeting [6,7].

Despite the effectiveness of these systems, their mode of action begs unanswered questions and leaves much to be understood. Indeed, the mechanisms whereby macromolecular drugs and nanoparticles offer advantages over conventional dosage forms were previously thought to be established. Such was the case of the enhanced permeability and retention (EPR) effect in the passive targeting of nanoparticles for cancer tumours [8,9,10], mostly by systemic delivery; it has been recently questioned as being more nuanced than previously thought, with several properties of the nanoparticles and patient characteristics influencing its effectiveness [6,7]. Multiple biological processes are thought to be at play when accounting for differences found in the EPR effect of different type of nanoparticles (e.g., interactions of proteins present in body fluids and formation of nanoparticles protein corona, and differences in extravasation and subsequent interaction with the tumour microenvironment). To make educated statements about the relevancy of the EPR of nanoparticles in a certain pathology, the properties of nanoparticles that influence the previously mentioned biological processes should be studied in detail, i.e., size, shape, surface makeup, charge, density, etc. Some of these biological processes can be studied in vitro, like the formation of protein corona [11]. Investigating other processes, like extravasation and tumour accumulation, can be more challenging and must be studied in or ex vivo models using magnetic resonance imaging or fluorescence microscopy. Another challenging aspect in studying the systemic delivery of nanoparticles are their circulation times and degradation and metabolic pathways. Nanoparticles injected into the bloodstream are mostly cleared by opsonization and the mononuclear phagocyte system in the liver, spleen, lungs, and bone marrow [12]. On the other hand, nanoparticles intended for transmucosal oral delivery (inherent greater patient compliance than any other administration route) are mainly used to enhance the bioavailability of drugs, particularly highly insoluble, non-Lipinski molecules, and delicate biological macromolecules (known also as “biologics”). They face a different set of challenges because of the acidic conditions encountered in the gastric tract and abundance of degradative enzymes as part of the digestive system [13]. The study of the release of drugs or other therapeutic molecules like siRNA from nanocarriers, and their bioimaging upon oral or systemic administration still face unmet challenges. Often, these gaps have to be addressed on a case-by-case basis. Similar to the study of the EPR effect of nanocarriers cellular uptake, drug release kinetics and the associated mechanistic aspects in vitro or in vivo can all be highly variable and dependent on the physicochemical characteristics of both drug and the nanocarrier, as well as the physiological context [14,15,16,17,18,19].

Förster resonance energy transfer (FRET) is the transfer of energy from an excited donor chromophore to an acceptor chromophore and occurs if the emission spectrum of the former is overlapping with the absorption spectrum of the latter, given their distance is below 10 nm and the molecules are in a certain steric orientation. It has been widely used to study conformation changes in proteins and has been called the ‘spectroscopic ruler’ [20]. More recently, FRET measurements have been applied to the in vitro and in vivo study of nanoparticle systems, to expand upon previously used simple fluorescent labelling [21,22,23,24,25]. FRET measurements with nanoparticles can determine whether they are still in their intact state or have been degraded, since FRET is rapidly lost when the proximity of donor and acceptor increases. This is advantageous when compared to simple fluorescent labelling, where it is often difficult to tell if the label is still associated with the nanoparticles or was released.

Nanoemulsions (NEs) are liquid sub-micron colloidal oil droplets dispersed in water (o/w) with a lipophilic oily core, usually stabilized by a surfactant covered interface with the aqueous phase. In drug delivery applications, NEs are mostly used to deliver lipophilic drugs with a low bioavailability loaded into their hydrophobic core, like previously mentioned polymeric micelles. Hydrophilic therapeutic large macromolecules like proteins or nucleic acids can also be loaded onto the shell of the system by adsorbing them. The o/w interface can be coated with a polymer too, usually by electrostatic interactions between the surfactant and an oppositely charged polyelectrolyte, giving rise to a nanocapsule (NC) system with different physicochemical properties. By coating nanoemulsions with a negative zeta potential with the cationic polymer chitosan (CS), NCs with a positive surface charge are obtained. Such chitosan-coated NCs are known to exhibit improved colloidal stability [26]. Besides, they are muco- [27] and bioadhesive towards negatively charged biological membranes [28]. These NCs have been used as a drug delivery platform for insulin [29], flavonoids that interfere with bacterial quorum sensing [30], poorly water-soluble capsaicin [31,32], or vaccines [33]. Despite the fact that these NCs have consistently shown efficacy as drug delivery carriers, the precise mechanistic aspects that govern the interactions with the body and cellular fate upon being administered, are not fully understood. 

This study aims to examine the labelling of CS NCs by FRET and perform a physicochemical characterization of these systems in comparison with unlabelled ones. FRET is studied using donor quenching and sensitized emission of the acceptor dye and compared between dyes in solution and dyes associated with NCs. FRET was measured in NCs that contain both donor and acceptor dyes and mixtures of NCs that contain either donor or acceptor to determine if intra-NC and inter-NC FRET occurred. As a model of NC disintegration, ethanol was used to dissolve NCs, and FRET, absorbance at 650 nm, and count rate were measured. Finally, we studied the in vitro degradation of NCs using pancreatin and found that protein corona formation affects their degradation rates, as probed by dynamic light scattering (DLS) measurements.

## 2. Materials and Methods

### 2.1. Materials

Chitosan HMC 70/5 (Batch No. 212-140311-02) with a degree of acetylation of 32% (as determined by ^1^H NMR) and *M*_W_ = 5.01 × 10^4^ g/mol and *I*p = 2.18 (as determined by SEC-HPLC with MALLS-DRI-IV multidetection in 0.3 M acetic acid/0.2 M sodium acetate buffer) was obtained from Heppe Medical Chitosan GmbH (Halle, Germany). Miglyol^®^ 812 (a neutral oil formed by esters of caprylic and capric fatty acids and glycerol) was kindly provided by Peter Cremer Oleo (Hamburg, Germany). The surfactant lecithin (Epikuron^®^ 145v, a phosphatidylcholine enriched fraction of soybean lecithin) was kindly supplied by Cargill Germany (Krefeld, Germany). Lipophilic dyes 3,3′-Dioctadecyloxacarbocyanine perchlorate (DiO), 1,1-Dioctadecyl-3,3,3,3-tetramethy-lindocarbo-cyanine iodide (DiI), 1,1′-dioctadecyl-3,3,3′,3′-tetramethy-lindodicarbocyanine, 4-chlorobenzenesulfonate salt (DiD) and 1,1-dioctadecyl-3,3,3,3-tetramethylindotricarbocyanine iodide (DiR) were purchased from Santa Cruz Biotechnology (Heidelberg, Germany). Pancreatin from porcine pancreas was purchased from Merck (Darmstadt, Germany), dissolved in PBS (pH = 7.4, Merck, Darmstadt, Germany) and clarified by centrifugation at 5000× *g* for 10 min. Afterwards, the supernatant was filtered through 0.45 µm syringe filters to avoid interference of insoluble protein with dynamic light scattering measurements. Bovine serum albumin was purchased from Merck (Darmstadt, Germany). MilliQ water with a resistivity of 18.2 MΩ at 25 °C and passed through a filter with a pore size of 0.22 µm was used throughout. All other reagents used were of analytical grade, if not specified otherwise.

### 2.2. Nanocapsule Preparation

NCs and NEs were prepared by the solvent displacement technique first described by Calvo et al. [34], with some modifications. Briefly, an organic phase was formed by dissolving 40 mg of lecithin in 1 mL of ethanol, followed by the addition of 125 μL of Miglyol^®^ 812 and lipophilic dyes DiO, DiI, DiD and/or DiR and adding ethanol up to 10 mL (in the original protocol of Calvo et al. [34], acetone is used in the organic phase instead of ethanol). This organic phase was immediately poured over 20 mL of the aqueous phase composed of a chitosan solution (0.5 mg/mL dissolved in water with 5% stoichiometric with 1 M HCl excess). NCs were formed spontaneously due to the organic solvents diffusion and Marangoni effects of the organic phase [35]. Finally, the ethanol and some of the water were evaporated at 40 °C under vacuum on a R-210 Rotavapor (Büchi Labortechnik GmbH, Essen, Germany) and the volume of the formulations was reduced to 10 mL. The association efficiency of dyes was determined using Vivaspin 2 ultrafiltration cartridge filters of polyethersulfone (PES) with a molecular weight cut-off of 30,000 kDa membrane purchased from Sartorius (Göttingen, Germany). To this end, NCs were diluted 1:100 in water and centrifuged through the ultrafiltration membranes at 4000× *g* for 10 min. Afterwards, absorbance scans were recorded of both the original dispersion and the filtrate (see details below).

### 2.3. Size, Count Rate and Zeta Potential Measurements

Nanocapsules were characterized on the basis of average size distribution, size polydispersity, count rate, and zeta potential. Particle size, polydispersion index, and count rate were determined by dynamic light scattering with non-invasive back scattering (DLS-NIBS) at a measurement angle of 173°. The autocorrelation functions were fitted with the default non-negative least squares (NNLS) fit to calculate the intensity size distribution plots and thus evaluate the Z-average diameter. For count rate, derived count rate values were used, which are independent from the used attenuator setting. The zeta potential was measured by mixed laser Doppler velocimetry and phase analysis light scattering (M3-PALS). A Malvern Zetasizer NanoZS (Malvern, UK) fitted with a red laser (λ = 632.8 nm) was used for both methods. The Zeta Sizer Software (v 7.12, Malvern, UK) was used to acquire and evaluate the data. Samples were diluted 1:100 before the measurements, and these were carried out in triplicate.

### 2.4. Absorbance and Fluorescence Measurements

Absorbance measurements were recorded using a JASCO V-630 spectrophotometer (Groß-Umstadt, Germany) with 1 mL Hellma quartz cells (Mühlheim, Germany) and a light path length of 10 mm. We used absorbance measurements at 650 nm, where absorbance of the dyes (DiO-DiI) was negligible to determine the presence of NCs by turbidity of the dispersion.

Fluorescence measurements were performed using a Tecan Safire Microplate reader (Crailsheim, Germany). Due to potentially variable distances and orientations of donor and acceptor molecules in the core of the NCs, the standard equations to calculate FRET efficiencies do not apply. Therefore, relative values for FRET were calculated as the FRET index by measuring the donor emission in the presence and absence of the donor using the following formula [36]:

(1) $$F_{I}=1-\frac{I_{DA}}{I_{D}},$$

with *F_I_* = FRET index, *I_DA_* = donor emission in presence of the acceptor and *I_D_* = donor emission in absence of the acceptor.

### 2.5. Enzymatic Degradation

Enzymatic degradation was studied using a constant concentration of NCs and varying the concentration of soluble fraction of pancreatin. Measurements were performed in PBS (pH = 7.4) by dissolving the indicated amount of enzyme in PBS and diluting the NCs 1:100 in the PBS-enzyme solution. For measurements studying the influence of protein corona on the enzymatic degradation of NCs by pancreatin, the NCs were diluted 1:100 in a PBS solution containing 4% BSA, except for the BSA^−^-control for 20 min while derived count rate was recorded. After 20 min (*t* = 0 min), pancreatin was added to the appropriate samples and the measurements continued for an additional 50 min. All derived count rate measurements were normalized to the timepoint *t* = −20 min to account for differences in derived count rate due to the presence or absence of BSA. 

## 3. Results and Discussion

### 3.1. Physicochemical Characterization of NCs

**Figure 1:**
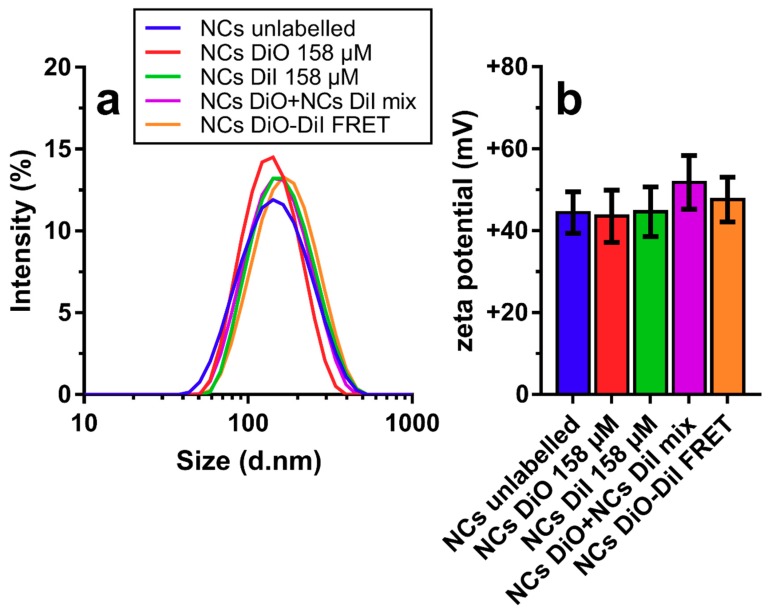
Physicochemical characterization of loaded and unloaded nanocapsules (NCs) by DLS-NIBS. (**a**) Scattering intensity over size; and (**b**) Zeta potential measured by diluting NC stocks 1:100 in water at 25 °C. Error bars in (**b**) represent standard deviation from 10 sub-runs used in the zeta potential measurements.

**Figure A1:**
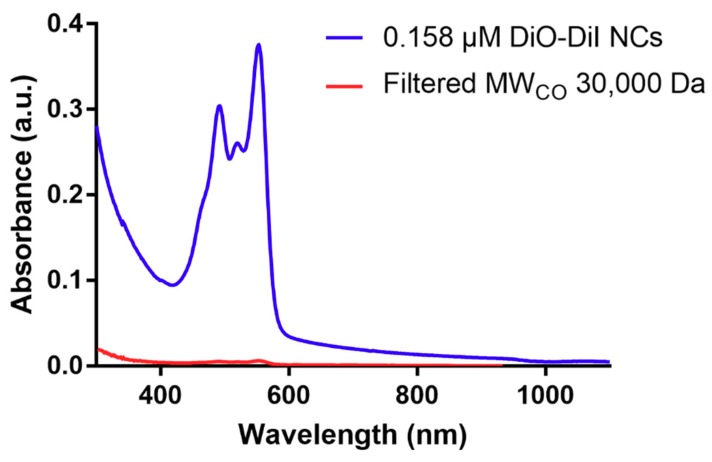
Absorbance scan of a 1:100 dilution of DiO-DiI NCs before (blue line) and after filtration (red line) through a 30,000 Da filter membrane for 10 min at 4000× *g*.

Figure 1 shows the physical characteristics, namely the hydrodynamic diameter distribution and average zeta potential, of unloaded CS-coated NCs, NCs loaded with DiO, DiI, a 1:1 mixture of both DiO-loaded NCs and DiI-loaded NCs, and individual NCs labelled with both dyes. Of note, there were almost no differences in the size distribution curves (Figure 1a), when labelled and unlabelled NCs was compared. The size of all different formulations oscillated around a Z-average hydrodynamic diameter of ~130 nm and zeta potential around ~+45 mV. However, mixing DiO- with DiI-loaded NCs resulted in a slightly elevated zeta potential (+50 mV), in comparison with their individual components; this increase could be related to the greater concentration of NCs in that sample. Regarding the association efficiency of the fluorescent dyes, we analysed the amount of unassociated fraction of each by centrifuging a DiO-DiI-NC dispersion over the PES membranes that prevented the NCs from passing through the membrane (see Figure A1). By dividing the peak absorbance for DiO and DiI after filtration by peak absorbance before filtration, we obtained association efficiencies of 98.28% and 98.37%, respectively. We found similar association efficiencies for closely related dyes (listed in Table A1), which allowed flexibility in labelling the near-infrared range.

The similarity between the Z-average size and zeta potential values of the developed dye-loaded NCs and those previously reported for NCs formulations using chitosans of varying *M_W_* (9572–279,200 g mol^−1^) and DA (1.6–57%) [26,31,32,34] suggests that the overall physical characteristics of these NCs seem to be hardly affected by the amount and nature of the compound encapsulated in the oil core [31], as confirmed in this study for dye molecules that are more lipophilic than the previously studied ones (*cf*., capsaicin; predicted partition coefficient (log P) DiO: 18.13 [37]; log P capsaicin: 3.81 [38]). The overall higher log P of the two examined dye molecules probably increased their capacity to associate within the oily core of the NCs to close to 100%, a value greater than the ~60–96% association efficiency previously determined for capsaicin-loaded NCs [31,32]. In previous studies, similar physicochemical characteristics examined by DLS were found for these nanocarriers, lacking fluorescent dyes, also studied the morphology and ultrastructure by transmission electron microscopy and synchrotron small-angle X-ray scattering (SAXS). The NCs were found to have spherical morphology with a core shell structure, and while the main driver of CS deposition on the surface was electrostatic interactions of CS and lecithin, SAXS revealed that hydrophobic interactions between hydrophobic moieties of CS and the lipid nucleus were also at play [31,32].

Strong affinity of the fluorescent dye for the NCs is a desired property, since this abrogates the need to isolate labelled NCs from free dye molecules. Although in this case, the extremely low water solubility of the dye molecules poses a challenge when trying to determine its release in vitro using completely aqueous environments. However, in experiments including cells in culture or in vitro (i.e., in the presence of cell membranes), the lipophilic dye molecules are readily able to associate with cell membranes upon release from the nanoparticles [22,25].

**Figure 2:**
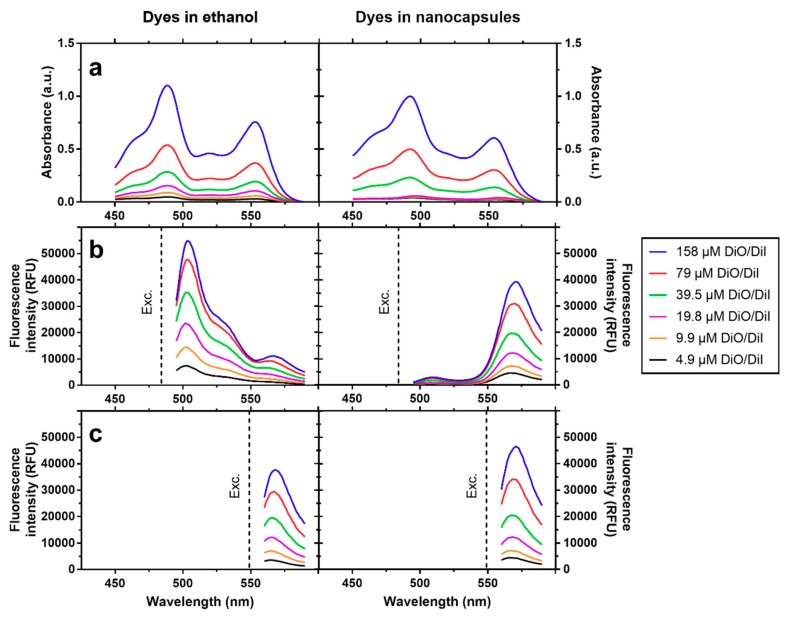
Spectroscopic properties of equimolar mixtures of DiO/DiI diluted in ethanol and equivalent concentrations of DiO/DiI in nanocapsules diluted in PBS (as shown in label). (**a**) Absorbance spectra. (**b**) Fluorescence emission spectra of DiO (donor), upon excitation (indicated by dotted line, λ_ex_ = 484 nm). (**c**) Fluorescence emission spectra of DiI upon excitation (indicated by dotted line, λ_ex_ = 549 nm).

Figure 2 shows a comparison of absorbance spectra and fluorescence emission spectra of equimolar mixtures DiO/DiI at varying total concentrations (between 4.9 µM and 158 µM) in ethanol on their own, and at similar concentrations and measured in PBS upon loading them in nanocapsules. The absorbance measurements have shown overall higher absorbance for the encapsulated dyes due to added turbidity by the NCs, although the dye peaks can easily be distinguished up to a dye concentration of 39.5 µM. Of note, when diluted in ethanol, upon exciting DiO (λ_ex_ = 484 nm), its fluorescence emission band (λ_em_ = 505 nm) clearly increased with concentration. In addition, a slight bleedthrough to the acceptor (DiI) was also noticed at λ = 565 nm (Figure 2b). By contrast, upon encapsulation, the DiO emission was almost completely quenched even at the highest concentration, while the acceptor emission strongly increased when compared with measurements in ethanol. Direct excitation of DiI (λ_ex_ = 549 nm) in both environments only showed slightly higher emission for the encapsulated dyes (Figure 2c).

The extinction measured by spectrophotometry of a NC dispersion is the combined effect of absorbance and scattering. In our case, this is due to dye molecules with strong absorbance in the visible spectrum and to the scattering caused by dispersed capsules. Given the sizes reported for the NCs above, scattering can be attributed to a mixture of Mie scattering (R_H_ ~ λ) and Rayleigh scattering (R_H_ ~ $\frac{1}{10}$λ) [39], assuming a previously reported spherical shape of the chitosan coated NCs [31]. From 450 to 590 nm, R_H_ is greater than $\frac{1}{10}$λ and Mie scattering predominates, meaning that all wavelengths of the visible spectrum are scattered equally. This results in a higher overall absorbance of the dilutions of the dyes in NCs compared to the dyes dissolved in ethanol. A strong fluorescence quenching is observed when exciting the donor in the presence of the acceptor in the case of NCs as compared to the dyes dissolved in ethanol, while the acceptor emission is almost unaffected when excited directly; this is an unequivocal hallmark of FRET at play. Due to the larger distance between dye molecules and overall greater mobility, no energy transfer can take place when dyes are diluted in ethanol, whereas the increased spatial proximity inside NCs (i.e., oil volume was 80-fold smaller compared to aqueous medium at the highest dye concentration) provided an environment for efficient energy transfer between dye molecules. FRET labelling of nanoscale systems using lipophilic tracer dyes, with a similar approach and findings as in this study, has been adopted to examine synthetic polymeric particles [25] and polymer coated and uncoated synthetic nanoemulsions [23,24].

**Figure 3:**
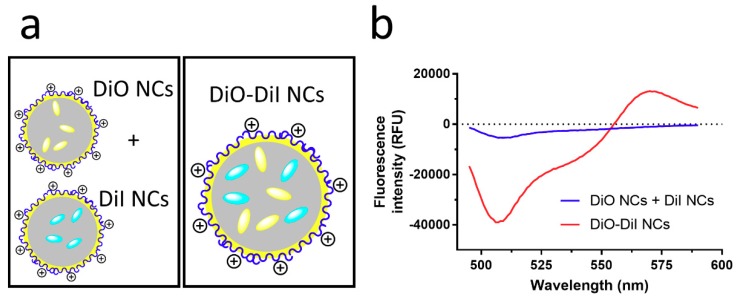
(**a**) Scheme of a mix of DiO- and DiI-loaded nanocapsules (NCs) and DiO/DiI-loaded NCs, and (**b**) Differential fluorescence spectra corrected by subtracting spectra of DiO NCs and DiI NCs from spectra of a physical blend of DiO- and DiI-loaded (0.158 µM) NCs and equimolar DiO/DiI-loaded NCs (0.158 µM) at the peak DiO excitation wavelength (λ_ex_ = 484 nm).

Figure 3 shows that upon mixing NCs containing either the donor or acceptor dyes, only a slight negative shift of the donor emission from that of the controls is obtained by measuring donor and acceptor NCs individually. By contrast, NCs containing both the donor and acceptor dyes display the previously shown behaviour of donor quenching and enhanced acceptor emission, deviating strongly from the spectra obtained for donor and acceptor NCs.

The slight negative emission observed for mixed donor- and acceptor-loaded NCs can be accounted for by the doubling in concentration of NCs needed to obtain the same concentration of both dyes in the dispersion. The NCs themselves scatter some of the incident and emitted light, while functioning as an inner filter, thus resulting in slightly lower fluorescence intensities [40]. The absence of FRET in this case indicates that the distance between capsules and hence, the distance between dye molecules, is too large (>10 nm) to establish inter-capsular FRET. When both dyes are loaded into the same NC, intra-capsular FRET is at play.

### 3.2. Dissolution and Enzymatic Degradation of NCs

**Figure 4:**
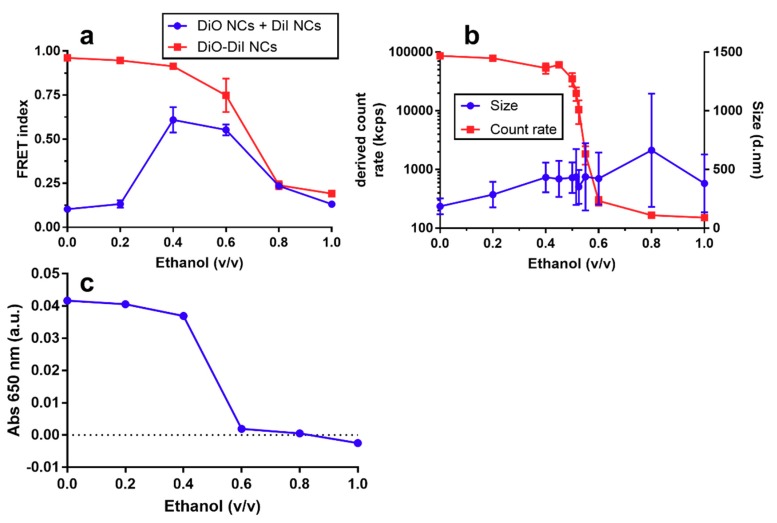
Effect of dissolution of fluorescently labelled nanocapsules (NCs) with increasing concentrations of ethanol, as characterized, by: (**a**) FRET index of a physical blend of DiO- and DiI-loaded (0.158 µM) NCs and equimolar DiO/DiI-loaded NCs (0.158 µM); (**b**) Derived count rate and Z-average size of equimolar DiO/DiI-loaded NCs (0.158 µM) by DLS-NIBS; and (**c**) Absorbance (λ = 650 nm) of equimolar DiO/DiI-loaded NCs. FRET index was calculated by measuring DiO (donor) emission in the presence and absence of DiI (acceptor) using Equation (1). Full spectra used to calculate FRET index are shown in Figure A1. Error bars represent standard deviation of triplicate measurements in all cases, except for size, where error bars represent mean PDI width in nm of triplicate measurements.

**Figure A2:**
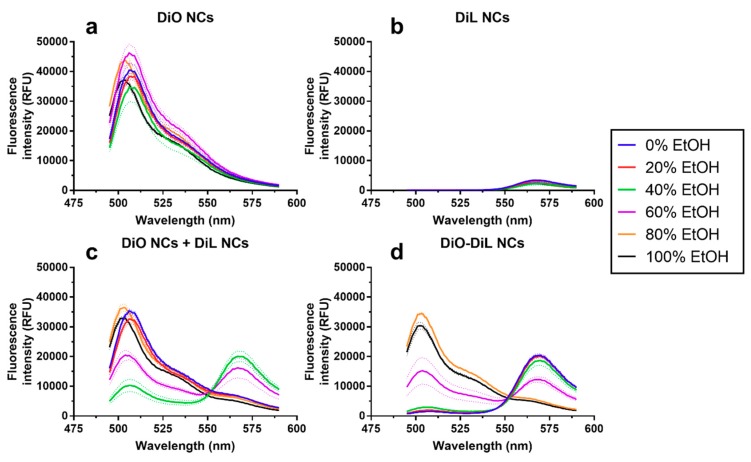
Emission spectra of (**a**) DiO NCs; (**b**) DiI NCs; (**c**) DiO NCs + DiI NCs and (**d**) DiO-DiI NCs at different Ethanol concentrations (*v*/*v* in water) upon excitation at λ = 484 nm.

The idea of FRET-labelling of drug delivery nanoparticles stems in the difficulty of studying their fate and the release of the payload when administered to cells in culture or in in vivo models. In this paper, we present an in vitro model to examine the degradation of NCs. For this purpose, we first dissolved dye-loaded NCs at different water/ethanol mixtures and measured their FRET index, absorbance at 650 nm, and derived count rate and particle size (Figure 4). The FRET index (F_I_), calculated by measuring the intensity of the donor in the presence of the acceptor divided by the intensity of the donor in absence of the acceptor (Equation (1)), showed a decrease of *F_I_* for the NCs that contain both dyes (donor and acceptor) in the same NCs, starting at ethanol concentrations higher than 0.4 (*v*/*v*) and decreasing further up to pure ethanol (Figure 4a). By contrast, physical blends of NCs that contained either donor or acceptor and showed no FRET in an aqueous environment (Figure 3b) exhibited FRET upon addition of ethanol at 0.4 and 0.6 (*v*/*v*), as shown in Figure 4a. In addition, the derived count rate (DCR) showed a sharp decrease with increasing ethanol/water ratios. Indeed, the DCR changed from 100,000 to 200 kcps upon addition of ethanol at ratios of 0.45 and 0.6 (*v*/*v*). This change was also accompanied by an increase in the Z-average sizes and PDI, albeit under a less-pronounced trend than the decrease in DCR. Absorbance (λ = 650 nm) also showed a step decrease at ethanol/water ratios (Figure 4c) of 0.4 to 0.6 (*v*/*v*). Additionally, for NCs containing only DiO, a slight shift of the DiO emission peak from 507 to 504 nm was observed from water to ethanol (Figure A2, Table A2).

The diminishing *F_I_* at increasing ethanol ratios observed for DiO/DiI-loaded NCs, alongside the change in the DiO peak emission wavelength and strongly decreasing count rate, is diagnostic evidence that the NCs are dissolved as the ethanol/water ratio attains a critical value between 0.4 to 0.6 (*v*/*v*). It was interesting to see that mixtures of DiO NCs and DiI NCs, which did not exhibit FRET in aqueous environments, started to exhibit a FRET at ethanol/water ratios >0.2 (*v*/*v*), with maximal *F_I_* values only slightly below those of DiO/DiI-loaded NCs. We suggest that this is the consequence of an increased solubility of the strongly hydrophobic dyes, originally trapped in the oily core of the NCs, in the ethanol/water mixed solvent and their resulting ability to interchange donor and acceptor dye molecules between NCs. Another possibility would be that the change in the overall polarity of the solvent brought by the addition of ethanol leads NCs to flocculate and coalesce, thus bringing donor and acceptor dyes into close contact. This proposal would be supported by the larger sizes attained with increasing ethanol ratios. The accompanying observed drastic drop in DCR at ethanol/water ratio of ~0.45 (*v*/*v*) could be interpreted as the result of the decrease in the overall number of coalescing particles. However, given that the size distribution measured by the intensity of light scattering by DLS-NIBS is sensitive to both the size and the overall number of particles, we can only offer a speculative interpretation to these results. Other complementary techniques, such as nanotracking analysis (NTA), which are amenable for characterizing the particle size distribution by number, may be informative in this regard in future studies.

The putative interexchange of dye molecules between NCs at intermediate ethanol/water compositions could have interesting implications on the behaviour of lipophilic molecules during the formation of the NCs using the solvent displacement technique. NCs are originally formed at a 0.33 ethanol/water ratio and from our results it seems feasible that loaded molecules, especially more hydrophilic molecules than the dyes used for this study, could still be exchanged between NCs. Release experiments in aqueous media often poorly predict the release of lipophilic drugs due to the lack of acceptors like cell membranes and lipid particles that are present in vivo. Previous studies have shown the use of single dye transfer between different lipophilic carriers like liposomes and lipid nanoparticles [41,42]. The use of dyes capable of FRET with different fat solubility or loading in separate NCs to study transfer kinetics could remove the need for separation by centrifugation or flow cytometry.

Fluorescence emission intensity and peak shifts of lipophilic dyes are frequently used to determine critical micellular concentrations (CMC) of surfactants [43,44]. Even when our experimental set-up was not designed to measure surfactant CMCs (e.g., of lecithin), the observed DiO emission blue shift could be taken as an indicator for a polarity change in its environment (i.e., a change from the strongly hydrophobic environment of the oily core of the NCs to the moderately hydrophobic environment of an ethanol-oil-lecithin solution). We did not observe a strong difference in fluorescence intensity between dye molecules associated with the NC oily core and NCs dissolved in ethanol, usually observed in DiO and related dyes [44]. Differences in fluorescence intensity are not expected to be as strong between the oily core and ethanol as previously reported differences between aqueous solutions and organic solvents because of a lower difference in polarity. The absence of emission intensity differences could also be due to an inner filter effect of the NCs quenching the incident and emission light.

**Figure 5:**
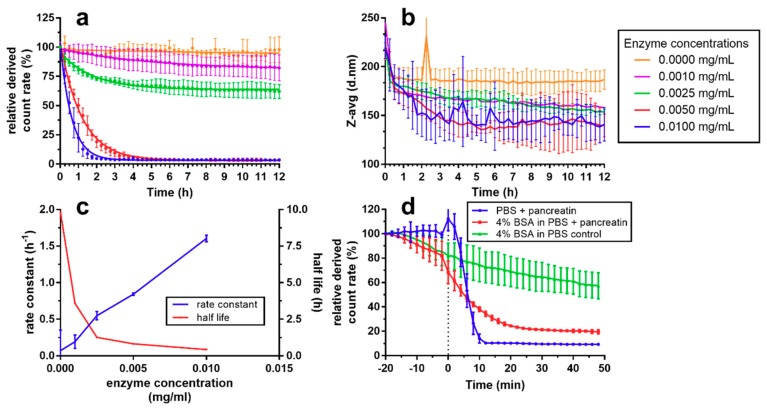
Enzymatic degradation of fluorescently labelled NCs using pancreatin measured by (**a**) derived count rate relative to initial value and (**b**) Z-avg particle size as measured by DLS. (**c**) Rate constant and half-life in dependence of enzyme concentration calculated from (**a**). (**d**) Degradation of CS NCs as diluted 1:100 in PBS and PBS with 4% bovine serum albumin as measured by derived count rate relative to initial values. Pancreatin was added at timepoint zero (dotted line) to a final concentration of 0.1 mg/mL for non-control samples. All measurements were performed at 37 °C (*n* = 3).

Figure 5 shows the results of the enzymatic degradation of NCs using pancreatin. Notice in Figure 5a,b that the normalized DCR size measurements were found constant when no pancreatin was present, whereas at higher enzyme concentration the degradation velocity increased in a linear fashion, as indicated by a linearly increasing rate constant between 0.001 and 0.01 mg/mL added pancreatin concentration. NC size decreased in samples containing pancreatin; greater the reduction the higher the pancreatin concentration is. However, there was a large overlap between concentrations. In a separate experiment, we determined the influence of a protein corona (BSA) on the degradability of the NCs (Figure 5d). Addition of 4% BSA yielded a slow decrease of DCR over 70 min, whereas in PBS without BSA, count rate was stable until pancreatin was added. After addition of pancreatin in samples without BSA, count rate was rapidly reduced to below 10% after 10 min. By contrast, the decrease was lower in samples containing BSA, reaching about 40% after 10 min, even though it had already decreased to about 80% before the addition of pancreatin due to BSA alone.

**Figure A3:**
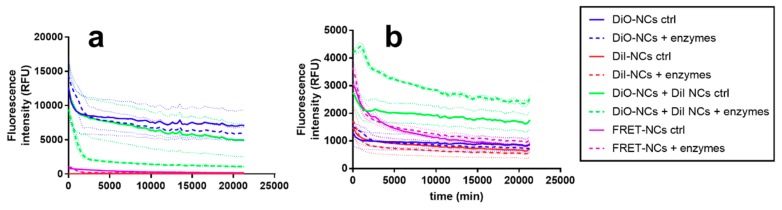
Emission spectra of fluorescently labelled nanocarriers at a. excitation at λ = 484 nm and emission at λ = 505 nm (donor excitation and emission) and b. excitation at λ = 484 nm and emission at λ = 568 nm (donor excitation and acceptor emission) over time at 37 °C. All nanocarriers were diluted to equivalent dye concentrations of 0.158 µM in PBS and samples that indicate addition of enzymes contained 0.1 mg/mL pancreatin. Small dashed lines above and below mean lines indicate standard deviation from three independent measurements.

Enzymatic digestion of nanoparticles is an important aspect to consider before their pre-clinical in vivo administration studies or clinical applications can be translated. This knowledge is necessary to ensure biocompatibility and full clearance of degraded components from the body’s tissues and prevent accumulation to toxic levels. Depending on the route of administration, nanoparticles will be in contact with different enzymes, pancreatin being an example of a mixture of enzymes present in the gastrointestinal tract relevant to oral administration. Pancreatin is a mixture of α-amylase, lipase, phospholipase and protease, which should be able to degrade all components of the NCs, except CS. In our experiments, however, we have shown that the CS-coated nanocapsules are readily degradable by pancreatin in PBS, as evidenced by the rapid reduction in DCR upon contact with the enzymes. Interestingly, the Z-average size did not decrease to zero even after 10 h, even when the DCR values reached their lowest level at a pancreatin concentration of 0.01 mg/mL. This could potentially be due to either a processive mode of enzymatic degradation, where there is a population of NCs that are destabilized and being degraded, while others remain intact, or a residual population of NCs that persists undegraded, which tend to be overestimated by the DLS measurements when compared to the decreasing size of NCs that are being degraded [45,46]. Unfortunately, unlike the dissolution of the NCs using ethanol, we were not able to probe the in vitro enzymatic degradation using the FRET approach presented in this paper, due to an overall decrease in fluorescence of both donor and acceptor upon enzymatic degradation of the NCs, presented in Figure A3. While a decrease of donor emission and increase in acceptor emission, both diagnostic hallmarks of FRET, were observed in the mixture of DiO NCs and DiI NCs that contained enzymes, over the control, other samples only showed an overall decrease in fluorescence. We attribute this to a probable photobleaching effect. In the mixture of NCs containing only one dye, NCs were probably destabilized and dyes were able to mix, similarly to the above discussed effects at intermediate ethanol concentrations. A lack of acceptors for lipophilic molecules in which the overall separation between dye molecules can increase upon release from the NCs is a limitation for in vitro experiments using FRET. Attempts to increase the solubility of the fluorescent dyes using surfactants failed due to inactivation of pancreatic enzymes. This could be addressed in future in vitro or in vivo experiments, given that cell membranes present could perform as acceptors for lipophilic dyes, as has been shown in previous studies [24].

The formation of a protein corona (PC) around nanoparticles is an expected phenomenon when they enter in contact with the proteins occurring in biological fluids. The influence of PC on pharmacokinetics, biodistribution, and biocompatibility has been of major interest recently [47,48,49,50,51]. Even though the combination of intestinal pancreatin and nanoparticles coated with a PC of BSA is an unrealistic in vivo scenario, simple in vitro models to elucidate the effects of protein adsorption on nanoparticles are needed to establish structure-function relationships prior to in vivo testing and clinical trials [48,52]. In our study, we found that adsorption of BSA slightly decreased the DCR of chitosan-coated NCs over time, probably due to an aggregation and sedimentation effect, given that NC size did not increase concomitantly. BSA was effective in forming a PC around NCs, which decreased accessibility of the NC to pancreatic enzymes, as detected by the lower degradation rates. We propose that DCR measurements recorded by DLS-NIBS are a readily accessible experimental parameter for quickly assessing either dissolution by solvents or enzymatic degradation of nanoparticles. Measurement of DCR allows a first screening of interactions of nanoparticles with model biological relevant environments, such as presence of pancreatic enzymes or BSA.

## 4. Conclusions

Altogether, our results have demonstrated that the labelling of CS-coated NCs using lipophilic tracer dyes did not affect their size distribution and zeta potential. When FRET donor (e.g., DiO) and acceptor (e.g., DiI) dye pair is co-loaded at equimolar ratios in NCs, the fluorescence emission of the donor dye is strongly quenched, whereas that of the acceptor dyes is enhanced when compared to the corresponding fluorescence of the dyes dissolved in ethanol. By contrast, no FRET was observed when dyes were loaded individually into NCs and mixed, thus indicating that FRET was only possible inside the oily core of the same NC, but not between different NCs. Dissolution of the NCs by partial addition of ethanol showed a decreasing FRET index for NCs loaded with both DiO and DiI. This was accompanied by a decrease in count rate and decrease in turbidity. On the other hand, physical blends of NCs loaded with either DiO or DiI under an identical treatment, first increased in FRET index and then decreased in a similar manner as DiO-DiI loaded NCs, which we interpret either as an exchange of lipophilic dye molecules at intermediate ethanol concentrations, before the complete dissolution of the NCs at high ethanol concentrations, or as the consequence of flocculation and coalescence of the nanoparticles. Lastly, we showed that the NCs were degraded by pancreatin in a dose-dependent manner and that a protein corona made from BSA decreased the rate of degradation. Ultimately, only further research, either in vitro or in vivo, will clarify the significance and allow to fully realize the application of the results presented here.

## Appendix A

**Table A1:** Excitation and emission maxima and predicted LogP for dyes encapsulated in NCs for this study.

Tracer Dye	Excitation Maximum (nm) ^1^	Emission Maximum (nm) ^1^	Log P ^2^
DiO	484	501	15.1
DiI	549	565	18.1
DiD	644	665	18.7
DiR	750	780	19.2

^1^ Data according to manufacturer manual. ^2^ Software prediction [37].

**Table A2:** Peak absorbance/peak emission of 1.59 µM DiO/DiI NCs (1:100 dilution) at different ethanol fractions in water (see full emission spectra in Figure A2).

Ethanol Fraction (*v*/*v*)	0	0.2	0.4	0.6	0.8	1	Δ0–1
DiO peak absorbance (nm)	491	491	491	488	488	487	4
DiO peak emission (nm)	507 ± 0.6	508 ± 1.7	507 ± 1.5	506 ± 0.0	504 ± 0.0	504 ± 0.6	3
DiI peak absorbance (nm)	552	553	553	550	551	551	1
DiI peak emission (nm)	567 ± 0.0	568 ± 1.0	569 ± 1.5	568 ± 1.2	566 ± 0.6	566 ± 0.6	1
